# Di-*n*-butyl 4,4′-dihy­droxy-3,3′-{[(3a*RS*,7a*RS*)-2,3,3a,4,5,6,7,7a-octa­hydro-1*H*-1,3-benzimidazole-1,3-di­yl]bis­(methyl­ene)}dibenzoate

**DOI:** 10.1107/S1600536811031205

**Published:** 2011-08-11

**Authors:** Augusto Rivera, Diego Quiroga, Jaime Ríos-Motta, Karla Fejfarová, Michal Dušek

**Affiliations:** a Departamento de Química, Universidad Nacional de Colombia, Ciudad Universitaria, Bogotá, Colombia; b Institute of Physics ASCR, v.v.i., Na Slovance 2, 182 21 Praha 8, Czech Republic

## Abstract

The complete molecule of the title compound, C_31_H_42_N_2_O_6_, is generated by crystallographic twofold symmetry, with one C atom lying on the axis. The dihedral angle between the aromatic rings is 57.03 (6)°. The central heterocyclic ring adopts a half-chair conformation. The mol­ecular conformation is stabilized by two intra­molecular O—H⋯N hydrogen bonds with the N atoms of the heterocyclic ring as the acceptors. In the crystal, mol­ecules are linked into chains along the *c* axis by non-classical C—H⋯O hydrogen bonds.

## Related literature

For related structures, see: Rivera *et al.* (2010 ▶, 2011 ▶); Giordano *et al.* (1999 ▶); Feng & Grant (2006 ▶). For background to this work see: Koll *et al.* (2001 ▶); Filarowski *et al.* (2004 ▶).
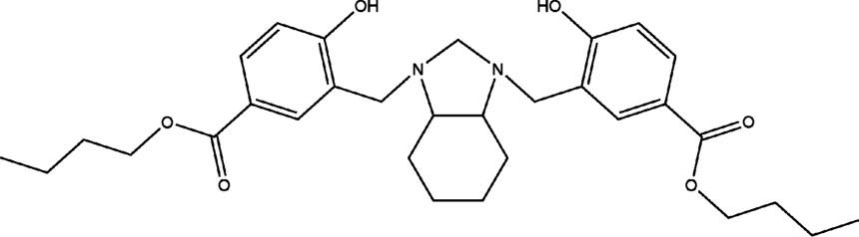


## Experimental

### 

#### Crystal data

C_31_H_42_N_2_O_6_
*M* *_r_* = 538.7
Monoclinic, 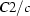

*a* = 15.4471 (3) Å
*b* = 8.8103 (2) Å
*c* = 20.9374 (4) Å
β = 95.077 (2)°
*V* = 2838.27 (10) Å^3^
*Z* = 4
Cu *K*α radiation
μ = 0.70 mm^−1^
*T* = 120 K
0.38 × 0.28 × 0.20 mm

#### Data collection

Agilent Xcalibur diffractometer with an Atlas (Gemini ultra Cu) detector
Absorption correction: multi-scan (*CrysAlis PRO*; Agilent, 2010 ▶) *T*_min_ = 0.868, *T*_max_ = 1.00
13938 measured reflections
2533 independent reflections
2157 reflections with *I* > 3σ(*I*)
*R*_int_ = 0.030

#### Refinement

*R*[*F* ^2^ > 2σ(*F* ^2^)] = 0.036
*wR*(*F* ^2^) = 0.104
*S* = 1.73
2533 reflections
181 parameters
H atoms treated by a mixture of independent and constrained refinement
Δρ_max_ = 0.20 e Å^−3^
Δρ_min_ = −0.19 e Å^−3^

### 

Data collection: *CrysAlis PRO* (Agilent, 2010 ▶); cell refinement: *CrysAlis PRO*; data reduction: *CrysAlis PRO*; program(s) used to solve structure: *SIR2002* (Burla *et al.*, 2003 ▶); program(s) used to refine structure: *JANA2006* (Petříček *et al.*, 2006 ▶); molecular graphics: *DIAMOND* (Brandenburg & Putz, 2005 ▶); software used to prepare material for publication: *JANA2006*.

## Supplementary Material

Crystal structure: contains datablock(s) global, I. DOI: 10.1107/S1600536811031205/bt5598sup1.cif

Structure factors: contains datablock(s) I. DOI: 10.1107/S1600536811031205/bt5598Isup2.hkl

Supplementary material file. DOI: 10.1107/S1600536811031205/bt5598Isup3.cml

Additional supplementary materials:  crystallographic information; 3D view; checkCIF report

## Figures and Tables

**Table 1:** Hydrogen-bond geometry (Å, °)

*D*—H⋯*A*	*D*—H	H⋯*A*	*D*⋯*A*	*D*—H⋯*A*
C5—H5*a*⋯O1^i^	0.96	2.39	3.2393 (16)	148
O3—H3⋯N1	0.884 (18)	1.873 (18)	2.6859 (12)	152.0 (17)

Symmetry code: (i) 
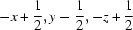
.

**Fig. 1.**
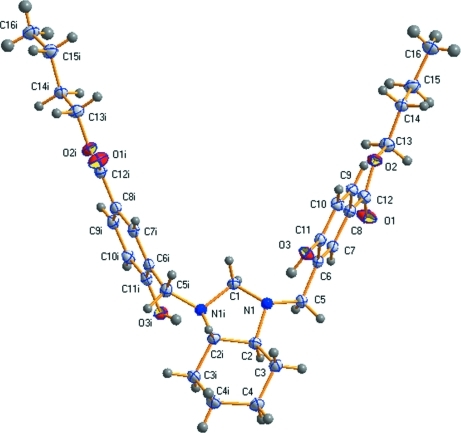
A view of (I) with the numbering scheme. Displacement ellipsoids are drawn at the 50% probability level.

**Fig. 2.**
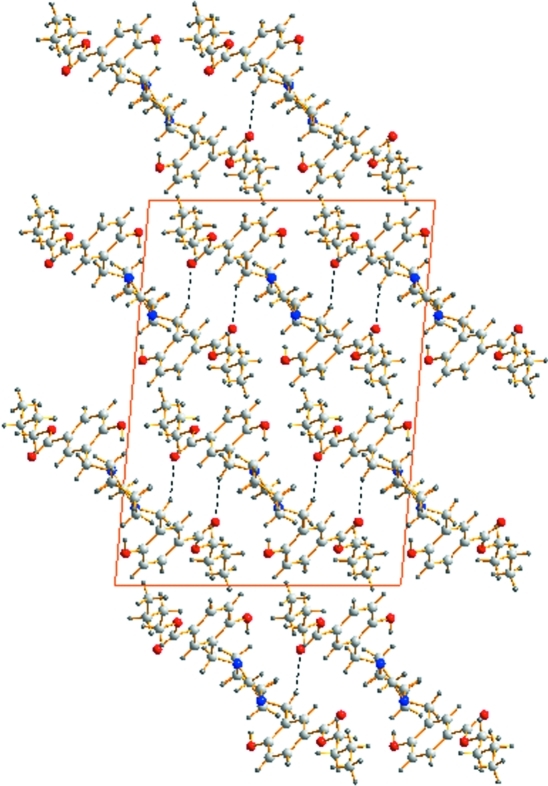
Packing of the molecules of the title compound view along b axis.

## supplementary crystallographic information

### Comment

Intramolecular hydrogen bonding interactions in Mannich bases lead to distortions in the aromatic rings due the existence of *ortho*-quinonoid resonance structures (Koll *et al.* 2001; Filarowski *et al.* 2004), which are evidenced in the bond and bond angle values. During our studies we have synthesized a series of symmetrical *di*-Mannich bases, by reaction between (2*R*,7*R*,11*S*,16S)-1,8,10,17-tetraazapentacyclo [8.8.1.1^8,17^.0^2,7^.0^11,16^]icosane and *p*-halophenols. The intramolecular hydrogen bond interaction is weaker in the Mannich base having *p*-fluorine substituent [N···O 2.711 (2) Å] (Rivera *et al.* 2011) whereas in the case of the Mannich base *p*-chlorine substituted [N··· O 2.652 (2) Å] (Rivera *et al.* 2010), due to electron-withdrawing effect of chlorine atom. However, the distortion in the aromatic ring in these Mannich bases, leads to a conclusion indicating that the effect of halogen atoms seems to be no meaningful which can be explained on the basis of the presence of conjugative electron-release from lone pairs of halogen atom. To avoid the conjugation effect of halogen to aromatic ring we synthesized the title compound (I) which is a racemic Mannich base with a non-halogen electron withdrawing group in *para* position. The molecular structure and atom-numbering scheme for (I) are shown in Fig. 1. The X-ray diffraction analysis shows the existence of two intramolecular hydrogen bonding interactions between the hydroxy H atom and the amine groups in the heterocyclic ring (table 1).

The observed C11—O3 bond length [1.358 (2)Å] is in good agreement with the *p*-chlorophenol derivative (Rivera, *et al.* 2010) but is shorter by 0.01 Å in comparison with *p*-fluorophenol derivative (Rivera, *et al.* 2011). Though, in the title compound the X-ray analysis revealed the existence of distortions of the phenol ring in comparison with the related structures (Rivera  *et al.* 2010, 2011), which was evident from the elongation of the C8—C9 bond length in title compound from [1.381 (2) Å, Rivera *et al.* 2011] to 1.393 (2) Å.

In comparison with the crystal packing of butylparaben, there are not head-to-tail hydrogen bonding interactions inside the chains that involve the O—H group as donor and the carbonyl O atom as acceptor (Giordano *et al.* 1999; Feng & Grant, 2006), there are non-classical hydrogen bonds C5—H5a··· O1 (table 1) which link neighboring helps stabilize the packing along *c* axis (Fig. 2).

### Experimental

A solution of (2*R*,7*R*,11*S*,16S)-1,8,10,17-tetraazapentacyclo [8.8.1.1^8,\17^.0^2,7^.0^11,16^] icosane (276 mg, 1.00 mmol) in dioxane (3 ml) and water (4 ml), previously prepared following described procedures, was added dropwise in a dioxane solution (3 ml) containing two equivalents of *n*-butyl 4-hydroxybenzoate (388 mg, 2.00 mmol) in a two-necked round-bottomed flask. The mixture was refluxed for about 12 h and then the solvent was evaporated under reduced pressure until a sticky residue appeared. The product was purified by chromatography on a silica column, and subjected to gradient elution with benzene:ethyl acetate (yield 19%, m.p. = 414–416 K). Single crystals of racemic (I) were grown from a chloroform solution by slow evaporation of the solvent at room temperature over a period of about 2 weeks.

### Refinement

All hydrogen atoms were discernible in difference Fourier maps and could be refined to reasonable geometry. According to common practice H atoms bonded C atoms were kept in ideal positions with C–H distance 0.96 Å during the refinement. The methyl H atoms were allowed to rotate freely about the adjacent C—C bonds. The hydroxyl H atoms were found in difference Fourier maps and their coordinates were refined freely. All H atoms were refined with displacement coefficients *U*_iso_(H) set to 1.5Ueq(C, O) for methyl and hydroxyl groups and to to 1.2Ueq(C) for the CH– and CH2- groups.

### Crystal data

**Table:**

C_31_H_42_N_2_O_6_	*F*(000) = 1160
*M**_r_* = 538.7	*D*_x_ = 1.260 Mg m^−^^3^
Monoclinic, *C*2/*c*	Cu *K*α radiation, λ = 1.5418 Å
Hall symbol: -C 2yc	Cell parameters from 7161 reflections
*a* = 15.4471 (3) Å	θ = 2.9–67.0°
*b* = 8.8103 (2) Å	µ = 0.70 mm^−^^1^
*c* = 20.9374 (4) Å	*T* = 120 K
β = 95.077 (2)°	Prism, colourless
*V* = 2838.27 (10) Å^3^	0.38 × 0.28 × 0.20 mm
*Z* = 4	

### Data collection

**Table:**

Agilent Xcalibur diffractometer with an Atlas (Gemini ultra Cu) detector	2533 independent reflections
Radiation source: Enhance Ultra (Cu) X-ray Source	2157 reflections with *I* > 3σ(*I*)
mirror	*R*_int_ = 0.030
Detector resolution: 10.3784 pixels mm^-1^	θ_max_ = 67.2°, θ_min_ = 4.2°
Rotation method data acquisition using ω scans	*h* = −17→18
Absorption correction: multi-scan (*CrysAlis PRO*; Agilent, 2010)	*k* = −10→10
*T*_min_ = 0.868, *T*_max_ = 1	*l* = −24→24
13938 measured reflections	

### Refinement

**Table:**

Refinement on *F*^2^	H atoms treated by a mixture of independent and constrained refinement
*R*[*F*^2^ > 2σ(*F*^2^)] = 0.036	Weighting scheme based on measured s.u.'s *w* = 1/[σ^2^(*I*) + 0.0016*I*^2^]
*wR*(*F*^2^) = 0.104	(Δ/σ)_max_ = 0.009
*S* = 1.73	Δρ_max_ = 0.20 e Å^−^^3^
2533 reflections	Δρ_min_ = −0.19 e Å^−^^3^
181 parameters	Extinction correction: B-C type 1 Lorentzian isotropic (Becker & Coppens, 1974)
0 restraints	Extinction coefficient: 2400 (500)
81 constraints	

### Special details

**Table:**

Experimental. CrysAlisPro (Agilent Technologies, 2010) Empirical absorption correction using spherical harmonics, implemented in SCALE3 ABSPACK scaling algorithm.
Refinement. The refinement was carried out against all reflections. The conventional *R*-factor is always based on *F*. The goodness of fit as well as the weighted *R*-factor are based on *F* and *F*^2^ for refinement carried out on *F* and *F*^2^, respectively. The threshold expression is used only for calculating *R*-factors *etc*. and it is not relevant to the choice of reflections for refinement.The program used for refinement, Jana2006, uses the weighting scheme based on the experimental expectations, see _refine_ls_weighting_details, that does not force *S* to be one. Therefore the values of *S* are usually larger than the ones from the *SHELX* program.

### Fractional atomic coordinates and isotropic or equivalent isotropic displacement parameters (Å^2^)

**Table:**

	*x*	*y*	*z*	*U*_iso_*/*U*_eq_	
O1	0.16850 (6)	0.28700 (12)	0.16508 (5)	0.0355 (3)	
O2	0.23006 (6)	0.43687 (10)	0.09549 (4)	0.0246 (3)	
O3	0.47351 (6)	−0.13568 (10)	0.08411 (4)	0.0251 (3)	
N1	0.45250 (4)	−0.23982 (9)	0.20231 (3)	0.0195 (3)	
C1	0.5	−0.13860 (11)	0.25	0.0198 (5)	
C2	0.45565 (8)	−0.39174 (13)	0.23189 (6)	0.0198 (4)	
C3	0.44634 (8)	−0.52666 (14)	0.18685 (6)	0.0254 (4)	
C4	0.45780 (9)	−0.67130 (15)	0.22757 (7)	0.0299 (4)	
C5	0.36225 (8)	−0.19088 (14)	0.18416 (6)	0.0224 (4)	
C6	0.35796 (8)	−0.04774 (14)	0.14442 (6)	0.0198 (3)	
C7	0.29735 (8)	0.06379 (14)	0.15343 (6)	0.0207 (4)	
C8	0.28954 (8)	0.19235 (14)	0.11452 (6)	0.0209 (4)	
C9	0.34430 (8)	0.20849 (15)	0.06556 (6)	0.0219 (4)	
C10	0.40580 (8)	0.09904 (15)	0.05580 (6)	0.0226 (4)	
C11	0.41305 (8)	−0.02913 (14)	0.09496 (6)	0.0208 (4)	
C12	0.22324 (8)	0.30676 (15)	0.12800 (6)	0.0227 (4)	
C13	0.16944 (8)	0.55491 (14)	0.11005 (7)	0.0258 (4)	
C14	0.17797 (8)	0.68569 (14)	0.06461 (6)	0.0235 (4)	
C15	0.12183 (9)	0.81927 (15)	0.08138 (7)	0.0289 (4)	
C16	0.12224 (10)	0.94748 (16)	0.03283 (7)	0.0347 (4)	
H1a	0.540883	−0.078171	0.22915	0.0238*	
H2	0.406857	−0.405328	0.256706	0.0237*	
H3a	0.490696	−0.522444	0.157548	0.0305*	
H3b	0.389436	−0.526022	0.164321	0.0305*	
H4a	0.408928	−0.683029	0.252453	0.0359*	
H4b	0.456989	−0.758307	0.19992	0.0359*	
H5a	0.332944	−0.174885	0.222158	0.0269*	
H5b	0.331366	−0.270607	0.16066	0.0269*	
H7	0.259748	0.052498	0.18726	0.0249*	
H9	0.339264	0.296369	0.038382	0.0263*	
H10	0.443506	0.111227	0.022094	0.0271*	
H13a	0.182864	0.588987	0.153344	0.0309*	
H13b	0.111248	0.515806	0.104655	0.0309*	
H14a	0.161007	0.653244	0.021541	0.0281*	
H14b	0.23765	0.717093	0.066364	0.0281*	
H15a	0.063247	0.785325	0.084261	0.0346*	
H15b	0.141765	0.856808	0.123154	0.0346*	
H16a	0.087619	1.030154	0.046201	0.0521*	
H16b	0.180815	0.981529	0.029994	0.0521*	
H16c	0.098561	0.911715	−0.00838	0.0521*	
H3	0.4785 (11)	−0.193 (2)	0.1188 (9)	0.0376*	

### Atomic displacement parameters (Å^2^)

**Table:**

	*U*^11^	*U*^22^	*U*^33^	*U*^12^	*U*^13^	*U*^23^
O1	0.0375 (6)	0.0326 (6)	0.0388 (6)	0.0083 (4)	0.0174 (5)	0.0107 (4)
O2	0.0237 (4)	0.0207 (5)	0.0297 (5)	0.0030 (3)	0.0044 (4)	0.0042 (4)
O3	0.0297 (5)	0.0239 (5)	0.0220 (5)	0.0055 (4)	0.0045 (4)	0.0015 (4)
N1	0.0209 (5)	0.0171 (5)	0.0198 (5)	0.0000 (4)	−0.0019 (4)	0.0003 (4)
C1	0.0220 (8)	0.0160 (8)	0.0210 (8)	0	−0.0007 (7)	0
C2	0.0217 (6)	0.0169 (6)	0.0204 (6)	−0.0003 (5)	0.0007 (5)	0.0012 (5)
C3	0.0275 (6)	0.0205 (7)	0.0270 (7)	−0.0003 (5)	−0.0044 (5)	−0.0025 (5)
C4	0.0348 (8)	0.0188 (7)	0.0345 (7)	−0.0021 (5)	−0.0062 (6)	−0.0024 (5)
C5	0.0191 (6)	0.0228 (7)	0.0246 (6)	−0.0008 (5)	−0.0016 (5)	0.0045 (5)
C6	0.0197 (6)	0.0207 (6)	0.0180 (6)	−0.0040 (4)	−0.0036 (5)	0.0005 (5)
C7	0.0186 (6)	0.0238 (7)	0.0194 (6)	−0.0032 (5)	−0.0001 (5)	0.0010 (5)
C8	0.0217 (6)	0.0200 (7)	0.0202 (6)	−0.0025 (5)	−0.0015 (5)	0.0007 (5)
C9	0.0266 (6)	0.0201 (6)	0.0186 (6)	−0.0030 (5)	−0.0004 (5)	0.0018 (5)
C10	0.0263 (6)	0.0239 (7)	0.0177 (6)	−0.0014 (5)	0.0030 (5)	0.0005 (5)
C11	0.0207 (6)	0.0218 (7)	0.0193 (6)	−0.0013 (5)	−0.0019 (5)	−0.0031 (5)
C12	0.0236 (6)	0.0234 (7)	0.0209 (6)	−0.0013 (5)	−0.0002 (5)	0.0028 (5)
C13	0.0230 (6)	0.0229 (7)	0.0320 (7)	0.0032 (5)	0.0055 (5)	0.0006 (5)
C14	0.0237 (6)	0.0209 (7)	0.0251 (7)	−0.0001 (5)	−0.0011 (5)	0.0003 (5)
C15	0.0264 (7)	0.0222 (7)	0.0382 (8)	0.0002 (5)	0.0039 (6)	−0.0005 (6)
C16	0.0362 (8)	0.0239 (7)	0.0432 (8)	0.0042 (6)	−0.0019 (6)	0.0033 (6)

### Geometric parameters (Å, °)

**Table:**

O1—C12	1.2100 (17)	C6—C7	1.3816 (17)
O2—C12	1.3421 (15)	C6—C11	1.4070 (17)
O2—C13	1.4498 (16)	C7—C8	1.3943 (17)
O3—C11	1.3575 (15)	C7—H7	0.96
O3—H3	0.884 (18)	C8—C9	1.3928 (18)
N1—C1	1.4836 (9)	C8—C12	1.4818 (18)
N1—C2	1.4738 (14)	C9—C10	1.3815 (18)
N1—C5	1.4765 (13)	C9—H9	0.96
C1—H1a	0.96	C10—C11	1.3941 (17)
C1—H1a^i^	0.96	C10—H10	0.96
C2—C2^i^	1.5061 (16)	C13—C14	1.5074 (18)
C2—C3	1.5163 (17)	C13—H13a	0.96
C2—H2	0.96	C13—H13b	0.96
C3—C4	1.5347 (18)	C14—C15	1.5213 (18)
C3—H3a	0.96	C14—H14a	0.96
C3—H3b	0.96	C14—H14b	0.96
C4—C4^i^	1.5381 (19)	C15—C16	1.520 (2)
C4—H4a	0.96	C15—H15a	0.96
C4—H4b	0.96	C15—H15b	0.96
C5—C6	1.5090 (17)	C16—H16a	0.96
C5—H5a	0.96	C16—H16b	0.96
C5—H5b	0.96	C16—H16c	0.96
			
C12—O2—C13	115.15 (10)	C8—C7—H7	119.2486
C11—O3—H3	105.7 (12)	C7—C8—C9	119.09 (11)
C1—N1—C2	105.49 (7)	C7—C8—C12	117.83 (11)
C1—N1—C5	113.40 (7)	C9—C8—C12	123.08 (11)
C2—N1—C5	111.64 (8)	C8—C9—C10	120.53 (11)
N1—C1—N1^i^	106.10 (7)	C8—C9—H9	119.7338
N1—C1—H1a	109.4715	C10—C9—H9	119.7345
N1—C1—H1a^i^	109.4713	C9—C10—C11	119.93 (12)
N1^i^—C1—H1a	109.4713	C9—C10—H10	120.0369
N1^i^—C1—H1a^i^	109.4715	C11—C10—H10	120.0376
H1a—C1—H1a^i^	112.6412	O3—C11—C6	120.83 (11)
N1—C2—C2^i^	101.91 (9)	O3—C11—C10	118.84 (11)
N1—C2—C3	116.92 (9)	C6—C11—C10	120.33 (11)
N1—C2—H2	110.0241	O1—C12—O2	122.61 (12)
C2^i^—C2—C3	110.19 (10)	O1—C12—C8	123.94 (12)
C2^i^—C2—H2	116.771	O2—C12—C8	113.44 (11)
C3—C2—H2	101.7125	O2—C13—C14	108.75 (11)
C2—C3—C4	107.78 (10)	O2—C13—H13a	109.4715
C2—C3—H3a	109.471	O2—C13—H13b	109.4713
C2—C3—H3b	109.4714	C14—C13—H13a	109.4714
C4—C3—H3a	109.4714	C14—C13—H13b	109.4715
C4—C3—H3b	109.472	H13a—C13—H13b	110.1822
H3a—C3—H3b	111.1071	C13—C14—C15	111.23 (11)
C3—C4—C4^i^	112.90 (11)	C13—C14—H14a	109.4707
C3—C4—H4a	109.4707	C13—C14—H14b	109.4712
C3—C4—H4b	109.4703	C15—C14—H14a	109.4716
C4^i^—C4—H4a	109.4719	C15—C14—H14b	109.4715
C4^i^—C4—H4b	109.4713	H14a—C14—H14b	107.6531
H4a—C4—H4b	105.8086	C14—C15—C16	112.60 (12)
N1—C5—C6	112.36 (10)	C14—C15—H15a	109.4712
N1—C5—H5a	109.4714	C14—C15—H15b	109.4717
N1—C5—H5b	109.4706	C16—C15—H15a	109.4711
C6—C5—H5a	109.4712	C16—C15—H15b	109.4712
C6—C5—H5b	109.4714	H15a—C15—H15b	106.1546
H5a—C5—H5b	106.42	C15—C16—H16a	109.4718
C5—C6—C7	121.09 (11)	C15—C16—H16b	109.4721
C5—C6—C11	120.22 (11)	C15—C16—H16c	109.4718
C7—C6—C11	118.62 (11)	H16a—C16—H16b	109.4713
C6—C7—C8	121.50 (11)	H16a—C16—H16c	109.4705
C6—C7—H7	119.2469	H16b—C16—H16c	109.4698
			
?—?—?—?	?		

Symmetry codes: (i) −*x*+1, *y*, −*z*+1/2.

### Hydrogen-bond geometry (Å, °)

**Table:**

*D*—H···*A*	*D*—H	H···*A*	*D*···*A*	*D*—H···*A*
C5—H5a···O1^ii^	0.96	2.39	3.2393 (16)	148.
O3—H3···N1	0.884 (18)	1.873 (18)	2.6859 (12)	152.0 (17)

Symmetry codes: (ii) −*x*+1/2, *y*−1/2, −*z*+1/2.

## References

[bb1] Agilent (2010). *CrysAlis PRO* Agilent Technologies, Yarnton, England.

[bb2] Brandenburg, K. & Putz, H. (2005). *DIAMOND* Crystal Impact GbR, Bonn, Germany.

[bb3] Burla, M. C., Camalli, M., Carrozzini, B., Cascarano, G. L., Giacovazzo, C., Polidori, G. & Spagna, R. (2003). *J. Appl. Cryst.* **36**, 1103.

[bb4] Feng, Y. & Grant, D. J. W. (2006). *Pharm. Res.* **23**, 1608–1616. 10.1007/s11095-006-0275-9 16783478

[bb5] Filarowski, A., Koll, A., Karpfen, A. & Wolschann, P. (2004). *Chem. Phys.* **297**, 323–332.

[bb6] Giordano, F., Bettini, R., Donini, C., Gazzaniga, A., Caira, M. R., Zhang, G. G. Z. & Grant, D. J. W. (1999). *J. Pharm. Sci.* **88**, 1210–1216. 10.1021/js9900452 10564071

[bb7] Koll, A., Parasuk, V., Parasuk, W., Karpfen, A. & Wolschann, P. (2001). *J. Mol. Struct.* **700**, 81–90.

[bb8] Petříček, V., Dušek, M. & Palatinus, L. (2006). *JANA2006* Institute of Physics, Praha, Czech Republic.

[bb9] Rivera, A., Quiroga, D., Ríos-Motta, J., Dušek, M. & Fejfarová, K. (2010). *Acta Cryst.* E**66**, o2643. 10.1107/S160053681003792X PMC2983180 21587614

[bb10] Rivera, A., Quiroga, D., Ríos-Motta, J., Dušek, M. & Fejfarová, K. (2011). *Acta Cryst.* E**67**, o1542. 10.1107/S1600536811019763 PMC3120376 21754901

